# Complete genome of *Microbacterium plantarum* CoE-159-22, isolated from traditionally produced Montenegrin cheese

**DOI:** 10.1128/mra.00035-24

**Published:** 2024-03-07

**Authors:** Beatriz Daza Prieto, Nadja Raicevic, Patrick Hyden, Tobias Mösenbacher, Johann Ladstätter, Anna Stöger, Adriana Cabal, Robert L. Mach, Aleksandra Martinovic, Werner Ruppitsch

**Affiliations:** 1 Institute of Medical Microbiology and Hygiene, Austrian Agency for Health and Food Safety, Vienna, Austria; 2 FoodHub—Centre of Excellence for Digitalization of Microbial Food Safety Risk Assessment and Quality Parameters for Accurate Food Authenticity Certification, University of Donja Gorica, Podgorica, Montenegro; 3 Institute of Chemical, Environmental, and Bioscience Engineering, Research Area of Biochemical Technology, Technical University of Vienna, Vienna, Austria; 4 Department of Biotechnology, University of Natural Resources and Life Sciences, Vienna, Austria; Indiana University, Bloomington, Indiana, USA

**Keywords:** food microbiology, whole-genome sequencing, Montenegro

## Abstract

*Microbacterium plantarum* (*M. plantarum*) was recently described as a new species isolated from copper globemallow (*Sphaeralcea angustifolia*). Here, we report the complete genome of *M. plantarum* CoE-159-22, which was obtained from traditionally produced Montenegrin cheese.

## ANNOUNCEMENT

The *Microbacterium plantarum* type strain was isolated from the inner tissue of leaves of copper globemallow [*Sphaeralcea angustifolia* (Cav.) G. Don] in Mexico and described as a new species in 2023 (1). It is closely related to *Microbacterium arborescens* that has been described as non-pathogenic and as a source of industrially important enzymes (2), used in food production (3).

*M. plantarum* CoE-159-22 was obtained from artisanal Montenegrin cheese according to ISO15214:1998 (4). CoE-159-22 was isolated on Listeria Chromogenic Agar according to Ottaviani and Agosti (ALOA) (Biomérieux, Austria). Species identification was performed by matrix-assisted laser desorption ionization-time of flight mass spectrometry (Microflex LT/SH system with MBT Compass IVD 4.1.100 module; Bruker, USA) and whole-genome sequencing.

Genomic DNA was isolated from colonies grown on blood agar with 5% sheep blood for 24 h/37°C/aerobiosis using the MagAttract high molecular weight DNA kit (Qiagen, Germany). DNA was neither sheared nor size selected prior to library preparations using the DNA Prep (M) kit (Illumina, USA) for 2 × 150 bp sequencing on a NextSeq 2000 (Illumina, USA) for short-read sequencing (5), the Native Barcoding Kit 24 V14 (SQK-NBD114.24, Oxford Nanopore Technology), and sequencing using a FLO-MIN114 (R10.4.1, Oxford Nanopore Technology) SpotON flow cell on a GridION according to the manufacturer’s instructions (Oxford Nanopore Technologies, UK) for long-read sequencing. A total of 70,214 Nanopore reads, with a N50 value of 10,990 bp, were obtained using dorado v7.1.4 in Superior base-calling mode and filtered using Filtlong v0.2.1 with the following parameters: min_length, 1,000; keep percent, 90; and target_bases, 500,000,000. A total of 3,484,180 Illumina reads were visually quality controlled using FastQC v0.11.9 (6) and used untrimmed in the final hybrid assembly using Unicycler v0.5.0 (https://github.com/rrwick/Unicycler.wiki.git), which resulted in 1 contig with a mean coverage of 149-fold, total length of 3,356,760 bp, and Guanine + Cytosine content of 69.7% (Table 1). Unicycler v0.5.0 reported the contig as circular.

**TABLE 1:** Characteristics of *Microbacterium plantarum* CoE-159-22^*a*^

Attribute	Finding
Assembly size (bp)	3,356,760
No. of contigs	1
No. of short reads	3,484,180
No. of long reads	70,214
Long-read fragment N50 (bp)	10,990
GC content (%)	69.7
Genome coverage	149
Total no. of genes	3,266
Total no. of coding sequences	3,195
No. of RNA genes	56
No. of tRNAs	47
ARG	*VanY* in *VanM* cluster (33% identity)
BAGEL4	Sactipeptides
AntiSMASH	Carotenoid, merochlorin A/B, deschloro-merochlorin A/B, isochloro-merochlorin B, dichloro-merochlorin B, merochlorin C/D, microansamycin, RRE-containing, thiopeptide, LAP, clifednamide, hygromycin

^*a*^ ARG, antimicrobial resistance gene.

The Comprehensive Antibiotic Resistance Database (7) and tools from the Center for Genomic Epidemiology (https://www.genomicepidemiology.org, 3rd December 2023) were used for detection of antimicrobial resistance genes, virulence genes, and plasmids. BAGEL4 (8) and antiSMASH v7.1.0 (9) were used to detect genes for the synthesis of secondary metabolites and aromatic compounds. Default parameters were used for all software unless otherwise specified.

CoE-159-22 was identified as *M. plantarum* using digital DNA-DNA hybridization (dDDH) (formula d4) (https://tygs.dsmz.de) and Average Nucleotide Identity (10) showing 90% (cut-off > 70% dDDH) and 98.48% (cut-off > 95%) identity to *M. plantarum* NE2HP2^T^ (Accession No. JAQZCF000000000), 81% and 97.43% identity to *M. arborescens* RCB1 (Accession No. QNUW00000000), and 41.7% and 90.29% to the *M. arborescens* DSM 20754^T^ (Accession No. QNUV00000000), respectively, suggesting that *M. arborescens* RCB1 should be re-classified as *M. plantarum*.

The NCBI Prokaryotic Genome Annotation v6.6 (11) identified 3,266 genes, 3,195 coding genes, 15 pseudogenes and 56 RNA genes (Table 1).

CoE-159-22 carried several genes putatively conferring antioxidative and antimicrobial activities (Table 1) like the recently described Merechlorins isolated from Streptomyces (12). Our strain carried a partial *vanY* in *vanM* cluster (Table 1) but was susceptible to vancomycin (0.75 µg/mL).

## Data Availability

This whole-genome shotgun project has been deposited in NCBI GenBank database under the accession number CP141117, BioProject accession number PRJNA1052001, and BioSample accession number SAMN38810176. This is the first version of this genome. The raw sequence reads have been deposited in the Sequence Read Archive under the accession number SRR27197670 and SRR27198275. Strain CoE-159-22 has been deposited in the DSMZ cell culture collection under the accession number DSM 117357.

## AFTER EPUB

*[This article was published on 7 March 2024 with the DSMZ number missing from the Data Availability paragraph. The Data Availability paragraph was updated in the current version, posted on 25 March 2024.]*
